# Emergency Department Use Across Income Groups Following an Increase in Cost-Sharing

**DOI:** 10.1001/jamanetworkopen.2023.29577

**Published:** 2023-08-17

**Authors:** Yushan Wu, Dorothy Yingxuan Wang, Shi Zhao, Maggie Haitian Wang, Eliza Lai-yi Wong, Eng-kiong Yeoh

**Affiliations:** 1 JC School of Public Health and Primary Care, The Chinese University of Hong Kong, Hong Kong Special Administrative Region, Hong Kong, China; 2 Centre for Health Systems and Policy Research, The Chinese University of Hong Kong, Hong Kong Special Administrative Region, Hong Kong, China; 3 Shenzhen Research Institute, The Chinese University of Hong Kong, Shenzhen, China

## Abstract

**Importance:**

To encourage the appropriate utilization of emergency care, cost-sharing for emergency care was increased from HK$100 (US $12.8) to HK$180 (US $23.1) per visit in June 2017 in all public hospitals in Hong Kong. However, there are concerns that this increase could deter appropriate emergency department (ED) visits and be associated with income-related disparities.

**Objective:**

To examine changes in ED visits after the fee increase.

**Design, Setting, and Participants:**

This retrospective cohort study used administrative data from June 2015 to May 2019 from all public hospitals in Hong Kong. Participants included all Hong Kong residents aged 64 years and younger, categorized into low-income, middle-income, and high-income groups according to the median household income in their district of residence. Data analysis was performed from May to June 2023.

**Main Outcomes and Measures:**

The primary outcome was the ED visit rate per 100 000 people per month, categorized into 3 severity levels (emergency, urgent, and nonurgent). Secondary outcomes include general outpatient (GOP) visit rate, emergency admission rate, and in-hospital mortality rate per month at public hospitals. Segmented regression analyses were used to estimate changes in the level and slope of outcome variables before and after the fee increase.

**Results:**

This study included a total of 5 441 679 ED patients (2 606 332 male patients [47.9%]; 2 108 933 patients [38.5%] aged 45-64 years), with 2 930 662 patients (1 407 885 male patients [48.0%]; 1 111 804 patients [37.9%] aged 45-64 years) from the period before the fee increase. The fee increase was associated with an 8.0% (95% CI, 7.1%-9.0%) immediate reduction in ED visits after June 2017, including a 5.9% (95% CI, 3.3%-8.5%) reduction in urgent visits and an 8.9% (95% CI, 8.0%-9.8%) reduction in nonurgent visits. In addition, a 5.7% (95% CI, 4.7%-6.8%) reduction of emergency admissions was found, whereas no significant changes were observed in in-hospital mortality. Specifically, a statistically significant increase in GOP visits (4.1%; 95% CI, 0.9%-7.2%) was found within the low-income group, but this association became insignificant after controlling for the social security group, who were exempted from payment, as a control.

**Conclusions and Relevance:**

In this cohort study, the fee increase was not associated with changes in ED visits for emergency conditions, but there was a negative and significant association with both urgent and nonurgent conditions across all income groups. Considering the marginal increase in public GOP services, further study is warranted to examine strategies to protect low-income people from avoiding necessary care.

## Key Points

**Question:**

Is increased cost-sharing associated with changes in emergency department (ED) visits for emergency, urgent, and nonurgent conditions?

**Findings:**

In this cohort study using administrative data for 5 441 679 ED patients at all public hospitals in Hong Kong, an increase in cost-sharing from HK$100 (US $12.8) to HK$180 (US $23.1) per ED visit was associated with a statistically significant reduction in urgent and nonurgent ED visits across all income groups, but no significant changes in overall emergency ED visits.

**Meaning:**

The statistically significant reduction in urgent ED care, particularly among the low-income group, warrants further study to assess the long-term health impact of the fee increase.

## Teaser

This cohort study examines changes in emergency department visits in Hong Kong after a fee increase in June 2017.

## Introduction

Patients often visit emergency departments (EDs) for minor or nonemergency reasons,^1,2,3,4^ which is costly and could contribute to ED overcrowding. Subsequent delays in care for critical cases resulting from overcrowding could cause clinically adverse events, such as preventable hospitalizations^5^ and mortality.^5,6,7^ Cost-sharing is one approach that has been proposed and implemented globally to minimize nonemergency visits^8,9,10,11,12,13^ on the basis of the premise that a modest financial burden could improve patients’ engagement in decision-making regarding their medical service usage and reduce inappropriate usage.^14^

Studies^15,16^ have shown that increased cost-sharing is widely associated with reduced ED use. In RAND’s landmark Health Insurance Experiment,^16^ people enrolled in a free care plan used 42% more ED services than those enrolled in the highest coinsurance plan. Higher coinsurance rates may create unintended decreases in both appropriate and inappropriate care consumption for individuals who are more ill and have low incomes, which may result in inferior health outcomes and inequities in access to care.^17^ Research^1,18,19^ has found cost-sharing for ED visits to be associated with reduced ED utilization among patients with severe conditions. Furthermore, low-income populations facing cost-sharing reduced their utilization more than higher-income populations did.^9,18^ However, previous research^1,9,15,18^ has primarily focused on privately insured patients, for whom evidence regarding the associations of cost-sharing with ED visits among low-income groups is limited and inconclusive.^8,20^ More research is needed to examine whether cost-sharing is a viable tool to strike a balance between appropriate and inappropriate ED care use without leading to income-related inequities.^21^

These issues are highly relevant in Hong Kong’s dual-track, tax-funded public health care system, which provides approximately 90% of inpatient services and 30% of outpatient services in the region while the private sector delivers the rest, funded by out-of-pocket payments and private insurance.^22^ Similar to the British National Health Service, public hospitals charge nominal fees that are inclusive of medications, laboratory tests, and basic procedures, and fees are waived for Comprehensive Social Security Assistance (CSSA) recipients (low-income people who are eligible for public financial assistance).^23^ Therefore, people with difficulty affording private outpatient care are more likely to visit EDs at public hospitals when medical needs arise,^24,25,26^ contributing to ED overcrowding and long wait times.^22^ Over the past few years, the Hospital Authority, which oversees all public hospitals in Hong Kong, has adopted various measures to manage the high demand for ED services, such as increasing staffing during influenza seasons.^24^

To address excessive ED visits, the Hospital Authority proposed an increase to ED fees in December 2016. The charge for each ED visit had previously remained unchanged at HK$100 (US $12.8) since 2002.^27,28^ After June 18, 2017, despite concerns that the charge increase may discourage low-income individuals from seeking timely medical care,^23^ the Hospital Authority increased the ED charge to HK$180 (US $23.1) per visit. However, after years of enactment, the economic and operational impact of this reform remains lacking without robust estimations.^29^

Hong Kong’s increase in cost-sharing for ED visits at public hospitals provides a unique opportunity to explore the association between cost-sharing and ED visits within a population. Our research aims to examine the changes in public ED care following the fee increase. It is worth mentioning that in the public ED setting, each patient undergoes assessment by experienced and specialty-trained nurses who prioritize treatment according to triage guidelines established since 1999. Regular audits ensure the quality of triage services,^30,31^ enabling us to analyze changes in ED visits by severity level. Although data on private care are unavailable, our public hospital data can potentially provide insights on changes in patients’ health care–seeking behavior and health outcomes following the increase in cost-sharing within the public sector.

Our primary objective was to examine whether the increase in cost-sharing for ED visits was associated with changes in overall public ED visits and the variation across different severity levels according to the triage system. Our secondary objective was to analyze the changes in general outpatient (GOP) visits, emergency admissions, and mortalities at public hospitals following the ED charge increase. Furthermore, we explored whether these changes exhibited any variations across different income groups.

## Methods

### Study Design

This cohort study used an interrupted time series design to examine changes in ED visits among the non-CSSA population that was subject to the fee increase for ED visits at public hospitals in Hong Kong effective June 2017. By use of data collected over time at regular intervals (eg, months), the underlying secular and postincrease trends can be distinguished while accounting for potential confounding factors that may influence the outcomes.^32^

The reporting of this study conforms to the Strengthening the Reporting of Observational Studies in Epidemiology (STROBE) reporting guidelines for cohort studies. The Survey and Behavioral Research Ethics Committee of the Chinese University of Hong Kong approved this study and waived the requirement for informed patient consent owing to the study’s use of deidentified data, in accordance with 45 CFR §46.

### Data

This study is primarily based on administrative data from the Hospital Authority between June 2015 to May 2019. The data set contains utilization records for all public health care services, including ED, GOP care, specialist outpatient care, and inpatient care. The data were aggregated by age group (0-14, 15-24, 25-44, 45-64, and ≥65 years), sex, district of residence, CSSA status, and month of attendance for each service type. We replaced all cells with values less than 5 with 2.5, because the data set did not include the exact number. This data set was further supplemented with population figures obtained from The Census and Statistics Department of Hong Kong.^33^

### Sample and Covariates

Our sample includes persons aged 64 years and younger. Older adults were excluded to rule out the impact of other concurrently introduced health care policies, such as fee-waiving schemes targeting individuals aged 65 years and older, that may confound the effects of the fee increase.^34,35^ District-level income variations have been observed historically in Hong Kong.^33,36^ As such, patients were categorized into 3 income groups according to the median household income in their respective districts of residence, with districts ranked by median income and evenly distributed across the groups.^37^ We included district-level age groups, sex, median district household income, and population size as covariates.

### Outcome Measures

Our primary outcome was the monthly total ED visit rates and visits by severity level (emergency, urgent, and nonurgent). ED patients undergo initial assessment by triage nurses and are classified into 5 categories (nonurgent, semiurgent, urgent, emergency, and critical) to determine the priority, intensity, and place of care (eTable 1 in Supplement 1).^31^ We collapsed the 5-level triage scale into 3 groups by combining semiurgent and nonurgent cases (levels 4 and 5) and critical and emergency cases (levels 1 and 2), respectively, because they have the same patient flow and treatment priority,^38^ and this provided the best overall validity.^30^ We calculated district-specific rates of ED visits per 100 000 people. Our secondary outcomes included monthly district-specific GOP visits rate, emergency hospital admissions rate, and in-hospital mortality rate per 100 000 people.

### Statistical Analysis

Data analysis was performed from May to June 2023 using Stata statistical software version 16 (StataCorp). Descriptive statistics for patient characteristics and outcome variables both before and after the fee increase in June 2017 were calculated. We then used segmented regression analyses to estimate changes in the level and slope of outcome variables before and after the fee increase, accounting for trends before June 2017.^39^ Because patients may react to the increase in cost-sharing immediately and adapt to it gradually over time, we hypothesized that there would be both a level change (an immediate change in outcome variables) and a slope change (a gradual change in the trend’s gradient) after the ED charge increase.^32^

We used a generalized linear regression with log link to specify the model for the outcome variables.^40^ Marginal effect sizes were estimated to quantify percentage changes in outcome variables before and after the policy change. We included time-varying district-level factors of age, sex, median household income, and modeled calendar month as a continuous variable to estimate the secular trend and included Fourier terms (pairs of sine and cosine functions) to address seasonal variation in health care utilization. We clustered SEs at the district level to correct for potential autocorrelation. We conducted subgroup analyses to examine whether changes in outcomes varied across the 3 income groups.

We conducted 3 sensitivity analyses. First, we excluded immediate postimplementation observations, assuming that it takes time for the news on the fee increase to reach the entire public. Here, immediate refers to the initial period following the implementation, where the associations may not yet be fully realized or widely known. Second, we replaced values less than 5 with 0 and 5, respectively, to examine how the estimates changed in the aggregated data set. Third, to assess the robustness of our findings in different income strata categorized by district-level median household income, we restricted our sample to the 3 lowest-income and highest-income districts and examined whether the estimates persisted across income groups.

We conducted 2 placebo tests. First, we replicated main analyses on CSSA recipients, who have no financial obligations for public services and should not have been affected by the fee increase. In addition, we conducted a difference-in-difference analysis to compare changes in outcome variables among the low-income group before and after the fee increase, using CSSA recipients as a control group. Second, we estimated our main model on nonemergency inpatient admissions and specialist outpatient care clinic visits, which necessitate referrals and are usually associated with lengthy waiting times in public hospitals. We assumed that these visits were not affected by the cost-sharing increase for ED visits in the short term. Statistical significance was set at 2-sided *P* < .05.

## Results

A total of 5 441 679 non–fee-waiving patients (2 606 332 male patients [47.9%]; 2 108 933 patients [38.5%] aged 45-64 years) visited EDs in public hospitals from June 2015 to May 2019 (Table 1). Among the 2 930 662 patients who visited before June 2017, 1 407 885 (48.0%) were male, 1 111 804 (37.9%) were aged 45 to 64 years, and 1 259 982 (43.0%) were from low-income districts. Visits were classified as emergency (70 873 visits [2.4%]), urgent (737 687 visits [25.2%]), and nonurgent (2 122 103 visits [72.4%]). Patient characteristics were comparable before and after June 2017.

**Table 1.** Characteristics of Patients Visiting Emergency Departments in Hong Kong Public Hospitals, June 2015 to May 2019^a^

Characteristic	Patients, No. (%)		
	All (N = 5 441 679)	June 2015-May 2017 (n = 2 930 662)	June 2017-May 2019 (n = 2 511 017)
Sex			
Male	2 606 332 (47.9)	1 407 885 (48.0)	1 198 447 (47.7)
Female	2 835 347 (52.1)	1 522 777 (52.0)	1 312 570 (52.3)
Age, y			
0-14	958 595 (17.6)	523 440 (17.9)	435 155 (17.3)
15-24	619 385 (11.4)	345 495 (11.8)	273 890 (10.9)
25-44	1 754 767 (32.2)	949 925 (32.4)	804 842 (32.1)
45-64	2 108 933 (38.8)	1 111 804 (37.9)	997 130 (39.7)
District-level income levels^b^			
High	1 234 613 (22.7)	680 536 (23.2)	554 077 (22.1)
Middle	1 818 698 (33.4)	990 145 (33.8)	828 553 (33.0)
Low	2 388 368 (43.9)	1 259 982 (43.0)	1 128 386 (44.9)
Triage category of emergency department visits			
Emergency condition	142 976 (2.6)	70 873 (2.4)	72 103 (2.9)
Urgent condition	1 421 472 (26.1)	737 687 (25.2)	683 785 (27.2)
Nonurgent condition	3 877 231 (71.3)	2 122 103 (72.4)	1 755 128 (69.9)

^a^ All Hong Kong residents are included in our study except Comprehensive Social Security Assistance recipients, who are exempted from paying for public health care services.

^b^ The patients were categorized into 3 income groups according to the median household income in their respective districts of residence. We rank districts by the median income level in 2017 and evenly classified into 3 groups.

Table 2 presents monthly total ED visit rates before June 2017 (mean [SD], 1970.7 [18.5] visits per 100 000 population) and after June 2017 (mean [SD], 1827.4 [19.8] visits per 100 000 population). After accounting for underlying trends, we estimated an immediate 8.0% (95% CI, 7.1% to 9.0%) reduction in the level of total ED visits rate after June 2017, representing a decline of 157.7 visits per 100 000 residents, but no significant slope change (0.03%; 95% CI, −0.3% to 0.3%) compared with trends before the fee increase. Furthermore, there were significant reductions in the levels of urgent ED visits (−5.9%; 95% CI, −8.5% to −3.3%) and nonurgent ED visits (−8.9%; 95% CI, −9.8% to −8.0%) associated with the ED charge increase. The model shows no significant change in the level of emergency ED visits rates, but a monthly reduction (−0.5%; 95% CI, −0.6% to −0.4%) was detected, corresponding to a monthly change of 0.2 visit per 100 000 population. The Figure presents the actual and estimated monthly ED visits rate from June 2015 to May 2019.

**Table 2.** Changes in Level and Slope of Monthly ED Visit Rates, GOP Visit Rates, Emergency Admissions Rate, and In-Hospital Mortality Rates After the Public ED Charge Increase in June 2017^a^

Type of visit	Monthly rate/100 000 population, mean (SD)		Changes before vs after June 2017, % (95% CI)	
	June 2015-May 2017	June 2017-May 2019	Level change^b^	Slope change^b^
Total ED visit rates	1970.7 (18.5)	1827.4 (19.8)	−8.0 (−9.0 to −7.1)^c^	0.03 (−0.3 to 0.3)
Emergency	48.1 (0.6)	52.8 (0.4)	−0.6 (−3.9 to 2.7)	−0.5 (−0.6 to −0.4)^c^
Urgent	496.1 (4.8)	497.6 (5.4)	−5.9 (−8.5 to −3.3)^c^	−0.3 (−0.5 to 0.01)^d^
Nonurgent	1426.9 (15.1)	1277.3 (14.5)	−8.9 (−9.8 to −8.0)^c^	0.1 (−0.2 to 0.4)
GOP visit rates	5671.9 (57.9)	5480.9 (77.7)	3.1 (0.9 to 5.3)^e^	−0.3 (−0.5 to −0.02)^d^
Emergency admissions rates	457.9 (5.7)	473.7 (4.9)	−5.7 (−6.8 to −4.7)^c^	−0.4 (−0.9 to 0.02)
In-hospital mortality rates	9.2 (0.1)	9.0 (0.1)	−2.5 (−12.5 to 7.4)	0.2 (−0.009 to 0.4)

Abbreviations: ED, emergency department; GOP, general outpatient.

^a^ All Hong Kong residents except Comprehensive Social Security Assistance recipients were included in the analysis. We used data from June 2015 to May 2019.

^b^ Estimated changes were based on generalized linear regression models with log link, with district-months as the unit of analysis, assessing the before and after level change in outcomes and the slope change after ED charge increase while accounting for the trend of preintervention period. The models accounted for district-level factors of sex, age groups (<15, 15-24, 25-44, and 45-64 years), median income level, and calendar months and Fourier terms to account for seasonality in outcomes. Robust SEs were used to account for the clustering of repeated monthly measures at the district level.

^c^ *P* < .001.

^d^ *P* < .05.

^e^ *P* < .01.

**Figure.**
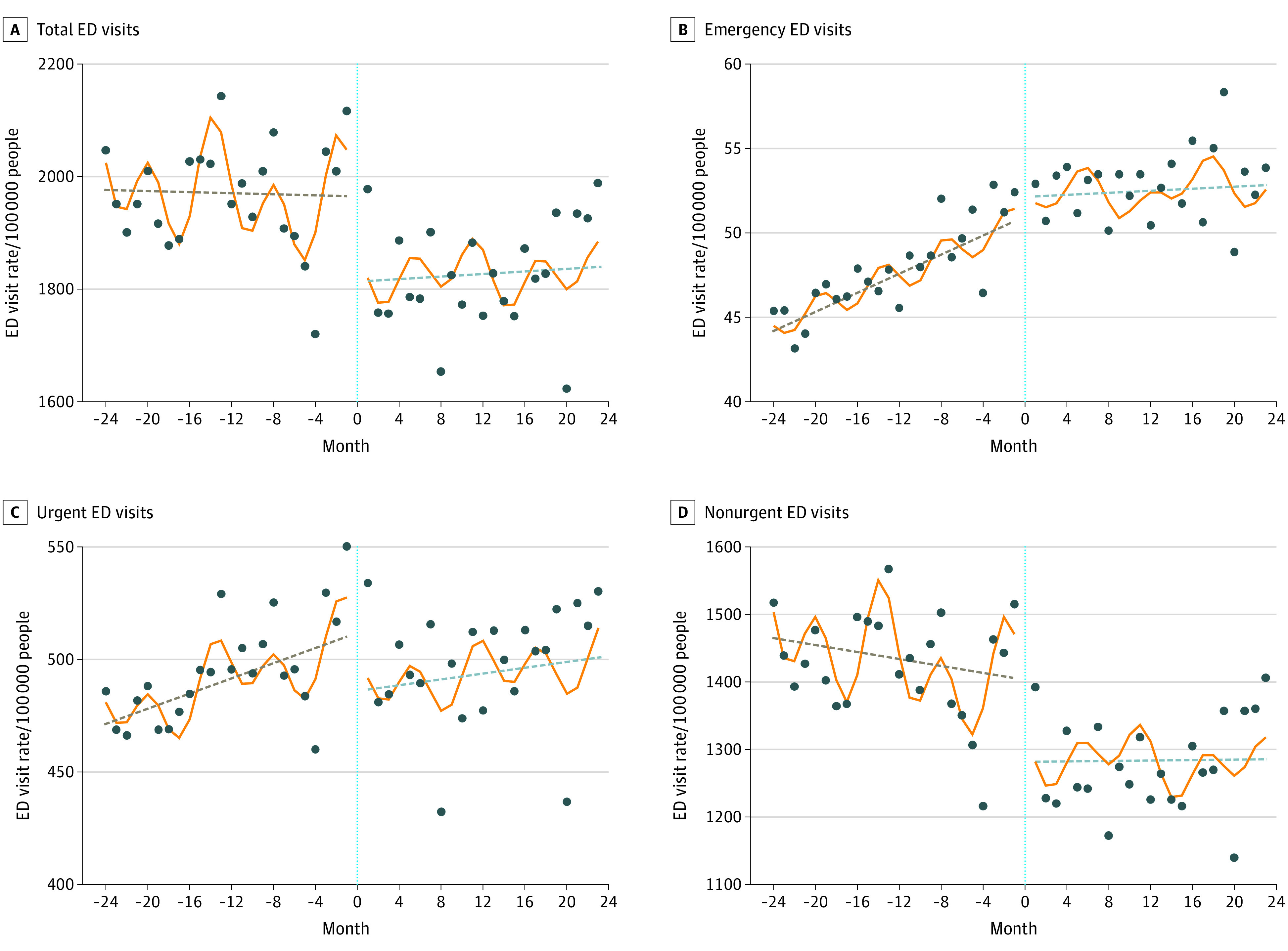
Monthly Emergency Department (ED) Visit Rates per 100 000 Population Among All Hong Kong Residents Except Those Enrolled in Comprehensive Social Security Assistance, June 2015 to May 2019 Graphs show total ED visits (A), emergency ED visits (B), urgent ED visits (C), and nonurgent ED visits (D). On the x-axes, the value of 0 represents June 2017, the time of increasing ED charge. The dots represent observed values. The solid lines represent the fitted estimates obtained using adjusted segmented regression, whereas the dashed lines depict the adjusted trends excluding month factors. All the fitted values were estimated using generalized linear regression models with a log link. The models accounted for district-level factors of sex, age groups (<15, 15-24, 25-44, and 45-64 years) and median income level. Additionally, calendar months and Fourier terms were included to account for seasonality in the outcomes.

Regarding secondary outcomes, we estimated a 3.1% (95% CI, 0.9% to 5.3%) increase in the level of GOP visits and a significant reduction of 5.7% (95% CI, 4.7% to 6.8%) in the level of emergency admissions after June 2017 using segmented regressions. No significant changes were detected in the level or slope of in-hospital mortality.

Table 3 shows that lower income districts exhibit higher monthly mean of ED visits rates, both in total and across triage categories. After June 2017, significant reductions in the level of urgent and nonurgent ED visits, as well as emergency admissions, were observed across all income groups. We found a significant increase in GOP visits (4.1%; 95% CI, 0.9% to 7.2%) only within the low-income group.

**Table 3.** Changes in Level and Slope of Monthly ED Visit Rates, GOP Visit Rates, Emergency Admission Rates, and In-Hospital Mortality Rates After the Public ED Charge Increase in June 2017, by District-Level Income Levels^a^

Income group and type of visit	Monthly rate/100 000 population, mean (SD)		Changes before vs after June 2017, % (95% CI)	
	June 2015-May 2017	June 2017-May 2019	Level change^b^	Slope change^b^
Low				
Total ED visit rates	2393.6 (26.0)	2321.1 (27.7)	−6.9 (−9.1 to −4.8)^c^	0.02 (−0.1 to 0.2)
Emergency	61.4 (0.7)	67.8 (0.8)	0.06 (−6.4 to 6.5)	−0.08 (−0.5 to 0.4)
Urgent	639.5 (5.7)	639.4 (7.0)	−5.1 (−7.2 to 3.1)^c^	−0.07 (−0.3 to 0.2)
Nonurgent	1693.4 (20.5)	1614.5 (21.0)	−7.9 (−11.6 to −4.2)^c^	0.05 (−0.2 to 0.3)
GOP visit rates	7182.8 (77.2)	6845.6 (98.8)	4.1 (0.9 to 7.2)^d^	−0.1 (−0.4 to 0.2)
Emergency admission rates	572.9 (6.5)	589.3 (6.3)	−7.8 (−14.7 to −0.9)^d^	−0.6 (−0.8 to −0.4)^c^
In-hospital mortality rates	12.9 (0.3)	12.3 (0.2)	−4.3 (−12.5 to 4.1)	0.1 (−0.1 to 0.4)
Middle				
Total ED visit rates	2225.7 (21.3)	2011.4 (22.1)	−9.4 (−12.2 to −6.5)^c^	−0.1 (−0.3 to 0.03)
Emergency	58.1 (0.6)	61.7 (0.6)	1.6 (−5.8 to 8.9)	−0.7 (−1.2 to −0.3)^e^
Urgent	549.3 (5.0)	541.2 (6.2)	−5.2 (−9.0 to −1.4)^e^	−0.4 (−0.7 to −0.1)^e^
Nonurgent	1619.1 (18.0)	1409.4 (16.2)	−10.7 (−14.7 to −6.7)^e^	−0.05 (−0.2 to 0.06)
GOP visit rates	5748.0 (60.0)	5533.4 (76.9)	2.7 (−0.1 to 5.5)	−0.05 (−0.3 to 0.2)
Emergency admission rates	493.8 (5.9)	514.1 (5.1)	−6.1 (−8.4 to −3.7)^e^	−0.6 (−1.2 to 0.07)
In-hospital mortality rates	11.7 (0.2)	11.3 (0.2)	−4.8 (−13.1 to 3.3)	0.4 (−0.1 to 0.9)
High				
Total ED visit rates	1747.6 (15.4)	1563.0 (14.8)	−7.4 (−9.9 to −4.9)^e^	0.2 (0.03 to 0.4)^d^
Emergency	42.5 (0.9)	48.4 (0.6)	−3.5 (−10.9 to 3.9)	−0.6 (−1.6 to 0.3)
Urgent	424.3 (5.5)	442.6 (4.7)	−6.3 (−11.0 to −1.6)^e^	−0.1 (−0.9 to 0.6)
Nonurgent	1282.4 (15.9)	1073.3 (10.7)	−8.1 (−11.2 to −5.0)^e^	0.3 (−0.002 to −5.0)^d^
GOP visit rates	4637.8 (47.2)	4500.0 (67.3)	2.1 (−2.0 to 6.4)	−0.6 (−1.3 to 0.001)
Emergency admission rates	474.1 (6.1)	485.0 (4.6)	−6.6 (−11.1 to −2.1)^e^	−0.5 (−1.0 to −0.04)^d^
In-hospital mortality rates	10.7 (0.2)	10.9 (0.2)	3.9 (−9.1 to 16.0)	0.13 (−0.07 to 0.3)

Abbreviations: ED, emergency department; GOP, general outpatient.

^a^ All Hong Kong residents except Comprehensive Social Security Assistance (CSSA) recipients were included in the analysis. Non-CSSA recipients were categorized into 3 income groups according to the median household income in their respective districts of residence. We rank districts by the median income level in 2017 and evenly classified into 3 groups.

^b^ Estimated changes were based on generalized linear regression models with log link, with district-months as the unit of analysis, assessing the before and after level change in outcomes and the slope change after ED charge increase while accounting for the trend of preintervention period. The models accounted for district-level factors of sex, age groups (<15, 15-24, 25-44, and 45-64 years), median income level, and calendar months and Fourier terms to account for seasonality in outcomes. Robust SEs were used to account for the clustering of repeated monthly measures at the district level.

^c^ *P* < .001.

^d^ *P* < .05.

^e^ *P* < .01.

### Sensitivity Analysis and Falsification Test

eTables 2 and 3 in Supplement 1 show that our estimates regarding the changes in the level of ED visit rates, emergency admission rate, and in-hospital mortality rate were robust to the exclusion of observations immediately after the policy change or adjusting cells of less than 5 to 5 or 0. Notably, the significant increase in GOP visits observed in Table 2 became insignificant when we excluded the 6-month observation period following the policy change. Furthermore, the changes in outcomes among high-income and low-income groups, as shown in eTable 4 in Supplement 1, were not qualitatively affected when we restricted the analysis to districts with the 3 highest and lowest median household incomes.

In placebo tests conducted for CSSA recipients, the fee-waiver group, we found no statistically significant change in outcome variables, except for GOP visits, which showed a significant increase (4.9%; 95% CI, 3.2% to 6.6%) before and after June 2017 (eTable 5 in Supplement 1). When using CSSA as a control, the statistically significant increase in GOP visits within the low-income group observed in Table 3 also became insignificant (eTables 6 and 7 in Supplement 1). In addition, eTable 8 in Supplement 1 indicates there were no significant changes in nonemergency admissions and specialist outpatient visits before and after the ED charge increase.

## Discussion

By use of administrative data from Hong Kong public hospitals, this cohort study found that the June 2017 fee increase was statistically significantly associated with an 8.0% decrease in overall ED visits rate, including a 5.9% decrease in urgent visits and an 8.9% decrease in nonurgent visits. No significant change was observed in the level of emergency ED visits. Subgroup analyses indicated that the reductions in urgent and nonurgent ED visits were statistically significant across all income groups. We also found a 5.7% reduction in the level of emergency admissions rate. Regarding short-term health outcomes, our analysis did not find any significant changes in in-hospital mortality rates. Furthermore, a significant increase in GOP visit rate was observed within the low-income groups after June 2017, but this increase became insignificant after using CSSA recipients as a control.

The higher reduction in nonurgent ED visits compared with urgent visits aligns with previous research^1,8,9,15,16^ suggesting that cost-sharing leads to greater reductions in inappropriate visits. However, the decline in urgent visits implies that some necessary ED care use may have also been discouraged in public hospitals.^41^ This finding raises concern about potential underuse of necessary health care,^42,43^ which could lead to detrimental health outcomes, as observed in the RAND Health Insurance Experiment.^44^ Previous research^11^ suggested that people may delay or forgo care because of the difficulty of determining the appropriateness of their ED visits when facing cost-sharing.

Furthermore, although the reduction in public ED visits may indicate decreased utilization, it is possible that individuals have opted for private or public outpatient care instead. Public GOP care in Hong Kong mainly serves patients who are older, have extremely low incomes, or have chronic disease.^41^ Our analysis did not show significant changes in public GOP visits in the middle-income and high-income groups. The observed increase in GOP visits both among the low-income and CSSA groups may be attributed to the increase of GOP quotas (the predetermined number of patients who can be seen in each GOP clinic) during the influenza season and long holidays from 2017 to 2019.^45^ It is also plausible that patients have shifted from public ED care to private care, seeking shorter waiting times and better perceived quality, particularly with the fee increase. However, the absence of private sector data precludes us from examining this further.

Although we observed significant reductions in urgent ED visits, we did not detect a statistically significant change in in-hospital mortality, indicating that no immediate adverse health outcomes were associated with the reduction in ED visits. Furthermore, the decrease in ED visits was accompanied by a significant reduction in the number of emergency admissions, similar to previous studies.^9,16,46^ This decrease may reflect a spillover effect of increased cost-sharing on reducing inappropriate hospitalization, because 29% of acute admissions via EDs in Hong Kong public hospitals were reported as being inappropriate.^47^ Nevertheless, these data must be interpreted carefully, because previous research^18^ suggests that a short-term decline in hospitalization might reflect forgone needed care rather than avoided unnecessary treatment. Further studies on long-term clinical outcomes of patients encountering cost-sharing for ED services are warranted.

Our study observed similar reductions in ED visits across 3 income groups. Previous studies^9,11,15,18^ in single-payer health systems generally reported greater declines in ED visits among low-income groups, indicating that these individuals are more price sensitive owing to decreased health care access, affordability issues, lower health literacy, and so forth.^19,48,49,50^ In the context of Hong Kong’s dual-track health care system, it is possible that low-income individuals may avoid seeking care in response to the increased fees, whereas high-income groups may opt for private services instead, leading to comparable reductions in public ED care as observed. However, the absence of private sector data precludes us from examining this premise and investigating whether the public fee increase exacerbated existing health care inequities.^51^ Furthermore, Hong Kong’s absence of a well-established primary care system may encourage overconsumption of ED resources among the low-income individuals.^52,53,54^ Because our income classification is based on district-level median income, it is possible that the observed ED visits primarily involved low-income individuals, even those residing in high-income districts. Additional studies are warranted to examine whether low-income groups avoided necessary care because of public charge increase and to explore remedial measures, such as reducing charges for urgent cases,^8,20^ providing subsidies for low-income groups,^18^ and, most important, improving primary care services.

### Limitations

Our study has several limitations. First, our analysis represents a fraction of the situation in Hong Kong given the absence of private sector data and deaths outside public hospitals. As such, although we were unable to quantify the extent of redirection or abandonment of reduced public ED use, our findings may still be inferable since public hospitals dominate inpatient care provision (especially severe cases), and most deaths occur in hospitals rather than in homes or other institutions.^55^ Second, our aggregated data precluded us from distinguishing between first and subsequent ED visits, which could have helped us gauge the diffusion of knowledge or awareness of the fee changes.^1^ Third, a retrospective study^31^ in Hong Kong public hospitals validated the Hong Kong Accident and Emergency Triage guidelines and found variation in internal validity among different triage categories. Nevertheless, our difference-in-difference analysis demonstrated consistent changes in ED visits by severity level, indicating that the triage assessments performed by designated nurses offer a degree of consistency in patient categorization.^30^ Fourth, our district-based measure of income is not equivalent to individual-level socioeconomic status. Nevertheless, our sensitivity analysis using the lowest-income and highest-income districts confirms our main analysis, suggesting the validity of our categorization of income levels based on district of residence.

## Conclusion

Hong Kong’s universal increase in cost-sharing for ED visits at public hospitals was associated with a reduction in nonurgent ED visits, suggesting that the government may have achieved its primary objective of reducing inappropriate ED visits. No changes in ED visits for emergency conditions were observed after the fee increase. However, the associated decrease in urgent ED visits, particularly among low-income groups, raises concerns about the potential avoidance of necessary care, especially when we observed no significant increase in public primary care. Further evaluation is necessary to assess the long-term clinical outcomes of patients encountering cost-sharing for public ED services.

## Notes

Supplement 1. **eTable 1.** Hong Kong Accident and Emergency Triage Guidelines **eTable 2.** Sensitivity Analysis Excluding 1 Month, 3 Months, and 6 Months of Observations Immediately After Policy Change **eTable 3.** Sensitivity Analysis Replacing Cells of ‘<5’ With ‘0’and With ‘5’ in Aggregated Dataset **eTable 4.** Sensitivity Analysis Restricting Samples to 3 Lowest- and Highest-Income Districts^a^ **eTable 5.** Placebo Analysis of Changes in Outcomes Among Comprehensive Social Security Assistance (CSSA) Patients **eTable 6.** Characteristics of Comprehensive Social Security Assistance (CSSA) Recipients and Low-Income Non-CSSA People Visiting Emergency Departments in Public Hospitals, 2015-2019^a^ **eTable 7.** Difference-in-Difference Analysis of the Changes in Outcomes Among Low-Income Groups, Using Comprehensive Social Security Assistance (CSSA) Patients as Control Group **eTable 8.** Placebo Analysis of Changes in Nonemergency Admissions and Specialist Outpatient Visits Before and After ED Charge Increase

Supplement 2.
Data Sharing Statement
