# Supplementary material for: Emergency Department Use Across Income Groups Following an Increase in Cost-Sharing
Source: JAMA Netw Open. 2023 Aug 17;6(8):e2329577. doi: 10.1001/jamanetworkopen.2023.29577 (PMC10436128; doi:10.1001/jamanetworkopen.2023.29577)
Supplement: Supplement 1. — eTable 1. Hong Kong Accident and Emergency Triage Guidelines eTable 2. Sensitivity Analysis Excluding 1 Month, 3 Months, and 6 Months of Observations Immediately After Policy Change eTable 3. Sensitivity Analysis Replacing Cells of ‘<5’ With ‘0’and With ‘5’ in Aggregated Dataset eTable 4. Sensitivity Analysis Restricting Samples to 3 Lowest- and Highest-Income Districtsa eTable 5. Placebo Analysis of Changes in Outcomes Among Comprehensive Social Security Assistance (CSSA) Patients eTable 6. Characteristics of Comprehensive Social Security Assistance (CSSA) Recipients and Low-Income Non-CSSA People Visiting Emergency Departments in Public Hospitals, 2015-2019a eTable 7. Difference-in-Difference Analysis of the Changes in Outcomes Among Low-Income Groups, Using Comprehensive Social Security Assistance (CSSA) Patients as Control Group eTable 8. Placebo Analysis of Changes in Nonemergency Admissions and Specialist Outpatient Visits Before and After ED Charge Increase [file jamanetwopen-e2329577-s001.pdf]

Supplemental Online Content

Wu Y, Wang DY, Zhao S, Wang MH, Wong ELY, Yeoh EK. Emergency department use across income groups following an increase in cost-sharing. *JAMA Netw Open*. 2023;6(8):e2329577. doi:10.1001/jamanetworkopen.2023.29577

eTable 1. Hong Kong Accident and Emergency Triage Guidelines

eTable 2. Sensitivity Analysis Excluding 1 Month, 3 Months, and 6 Months of Observations Immediately After Policy Change

eTable 3. Sensitivity Analysis Replacing Cells of ‘<5’ With ‘0’and With ‘5’ in Aggregated Dataset

eTable 4. Sensitivity Analysis Restricting Samples to 3 Lowest- and Highest-Income Districts[a]

eTable 5. Placebo Analysis of Changes in Outcomes Among Comprehensive Social Security Assistance (CSSA) Patients

eTable 6. Characteristics of Comprehensive Social Security Assistance (CSSA) Recipients and Low-Income Non-CSSA People Visiting Emergency Departments in Public Hospitals, 2015-2019[a]

eTable 7. Difference-in-Difference Analysis of the Changes in Outcomes Among Low-Income Groups, Using Comprehensive Social Security Assistance (CSSA) Patients as Control Group

eTable 8. Placebo Analysis of Changes in Nonemergency Admissions and Specialist Outpatient Visits Before and After ED Charge Increase

This supplemental material has been provided by the authors to give readers additional information about their work.

© 2023 Wu Y et al. *JAMA Network Open.*

eTable 1. Hong Kong Accident and Emergency Triage Guidelines

Triage category	Definition	Priority of care (% of patients required to fulfill the target response time)	Intensity of care	Place of care
1: Critical	A life-threatening condition caused by a major event<br>Unstable vital signs	Immediate medical attention (100%)	Team approach by physicians and nurses	Resuscitation area
2: Emergency	A potentially life-threatening condition<br>Borderline vital signs but with potential risk of rapid deterioration	Medical attention within 15 minutes (95%)	Immediate continuous close monitoring	Resuscitation area or treatment cubicle
3: Urgent	A major condition with potential risk of deterioration<br>Vital signs stable	Medical attention within 30 minutes (90%)	Nurse-initiated intervention as necessary	Cubicle
4: Semi-urgent	An acute but stable condition with minimal immediate risk of serious complication<br>Vital signs stable	-	-	Cubicle or walk-in clinic
5: Non-urgent	A minor and stable condition with no serious complication expected<br>Vital signs stable	-	-	Walk-in clinic

© 2023 Wu Y et al. *JAMA Network Open.*

eTable 2. Sensitivity Analysis Excluding 1 Month, 3 Months, or 6 Months of Observations Immediately After Policy Change

	Monthly mean, per 100 000 population (SD)		Percentage changes, after vs before June 2017	
	June 2015-May 2017	June 2017 -May 2019	Level Change[a] (95% CI)	Slope Change[a] (95% CI)
**Excluding 1 months of observations after change**				
Total ED visits	1969.3 (19.2)	1820.5 (19.4)	-10.1% (-12.9% to -7.3%)***	0.2% (0.1% to 0.3%)**
Emergency	47.9 (0.5)	52.8 (0.4)	-1.5% (-4.0% to 1.0%)	-0.4% (-0.7% to -0.2%)***
Urgent	494.3 (4.7)	496.0 (5.4)	-7.7% (-12.1% to -3.2%)**	-0.1% (-0.2% to -0.04%)**
Non-urgent	1427.6 (15.8)	1272.1 (14.1)	-11.1% (-13.3% to -8.8%)***	0.3% (0.1% to 0.5%)***
GOP visits	5663.4 (59.8)	5473.0 (80.9)	3.2% (0.4% to 6.0%)*	-0.3% (-0.5% to -0.1%)*
Emergency admissions	448.9 (5.5)	465.0 (4.9)	-7.0% (-10.4% to -3.6%)***	-0.3% (-0.6% to -0.1%)*
In-hospital deaths	9.2 (0.1)	9.0 (0.1)	-3.5% (-11.8% to 4.8%)	0.3% (0.1% to 0.5%)*
**Excluding 3 months of observations after change**				
Total ED visits	1969.3 (19.2)	1826.8 (20.9)	-10.1% (-12.8% to -7.4%)***	0.1% (0.05% to 0.3%)*
Emergency	47.9 (0.5)	52.9 (0.5)	-1.9% (-4.3% to 5.4%)	-0.4% (-0.7% to -0.2%)**
Urgent	494.3 (4.7)	497.3 (5.9)	-7.8% (-12.6% to -2.9%)**	-0.1% (-0.15% to -0.06%)***
Non-urgent	1427.6 (15.8)	1277.0 (15.1)	-10.9% (-13.0% to -8.7%)***	0.3% (0.1% to 0.5%)**
GOP visits	5663.4 (59.8)	5430.7 (83.2)	1.2% (-1.8% to 4.3%)	-0.1% (-0.3% to 0.05%)
Emergency admissions	448.9 (5.5)	465.7 (5.4)	-7.6% (-11.2% to -4.0%)***	-0.3% (-0.6% to -0.02%)*
In-hospital deaths	9.2 (0.1)	9.0 (0.1)	-3.2% (-10.4% to -4.1%)	0.2% (-0.01% to 0.5%)
**Excluding 6 months of observations after change**				
Total ED visits	1969.3 (19.2)	1828.2 (24.1)	-11.6% (-1.3% to -9.0%)***	0.3% (0.2% to 0.4%)***
Emergency	47.9 (0.5)	52.9 (0.5)	-2.3% (-10.1% to 5.6%)	-0.4% (-0.9% to 0.1%)
Urgent	494.3 (4.7)	497.5 (6.9)	-8.8% (-14.1% to -3.4%)**	-0.05% (-0.1% to 0.04%)
Non-urgent	1427.6 (15.8)	1278.1 (17.4)	-12.7% (-14.8% to -10.5%)***	0.4% (0.2% to 0.6%)***
GOP visits	5663.4 (59.8)	5420.5 (94.7)	3.0% (-1.2% to 7.1%)	-0.2% (-0.5% to -0.008%)*
Emergency admissions	448.9 (5.5)	467.0 (6.1)	-8.7% (-13.2% to -4.2%)***	-0.2% (-0.5% to 0.04%)
In-hospital deaths	9.2 (0.1)	9.1 (0.1)	-0.7% (-6.8% to 5.3%)	0.1% (-0.2% to 0.4%)

Abbreviation: SD, standard deviation; ED, emergency department; GOP, general outpatient.

a. Estimated changes were based on generalized linear regression models with log link, with district-months as the unit of analysis, assessing the pre-post level change in outcomes and the slope change after ED charge increase while accounting for the trend of preintervention period. The models accounted for district-level factors of sex, age groups (<15, 15-24, 25-44, 45-64 years), median income level, and calendar months and Fourier terms to account for seasonality in outcomes. Robust SEs were used to account for the clustering of repeated monthly measures at the district level.

***, *P*-value <0.001; **, *P*-value <0.01; *, *P*-value <0.05.

© 2023 Wu Y et al. *JAMA Network Open.*

eTable 3. Sensitivity Analysis Replacing Cells of ‘<5’ With ‘0’and With ‘5’ in Aggregated Dataset

	Monthly mean, per 100 000 population (SD)		Percentage changes, after vs before June 2017	
	June 2015-May 2017	June 2017 -May 2019	Level Change[a] (95% CI)	Slope Change[a] (95% CI)
**Replacing cells of ‘<5’ with ‘5’ in aggregated dataset**				
Total ED visits	2192.3 (18.6)	2047.0 (20.1)	-7.0% (-8.1% to -5.9%)***	0.01% (-0.3% to 0.3%)
Emergency	82.4 (0.8)	89.1 (0.6)	-0.2% (-4.0% to 3.7%)	-0.5% (-0.6% to -0.3%)***
Urgent	582.4 (4.9)	583.3 (5.5)	-5.1% (-8.1% to -2.1%)**	-0.2% (-0.4% to 0.02%)
Non-urgent	1528.2 (15.1)	1375.2 (14.6)	-8.1% (-8.6% to -7.6%)***	0.1% (-0.2% to 0.4%)
GOP visits	5678.6 (57.9)	5488.9 (77.7)	3.1% (0.9% to 5.3%)**	-0.3% (-0.5% to -0.03%)*
Emergency admissions	534.5 (5.9)	549.6 (5.0)	-4.8% (-6.3% to -3.3%)***	-0.4% (-0.8% to 0.04%)
In-hospital deaths	24.5 (0.2)	23.8 (0.3)	-4.6% (-14.1% to 4.8%)	0.1% (-0.009% to 0.3%)
**Replacing cells of ‘<5’ with ‘0 in aggregated dataset**				
Total ED visits	1749.1 (18.4)	1607.8 (19.5)	-9.8% (-11.9% to -7.6%)***	0.1% (-0.3% to 0.4%)
Emergency	13.8 (0.3)	16.6 (0.3)	-8.1% (-16.6% to 0.4%)	-1.3% (-2.2% to -0.5%)**
Urgent	409.6 (4.8)	411.9 (5.3)	-7.9% (-9.4% to -6.3%)***	-0.3% (0.6% to 0.02%)
Non-urgent	1325.7 (15.2)	1179.4 (14.4)	-10.1% (-12.2% to -8.1%)***	0.2% (-0.2% to 0.5%)
GOP visits	5663.5 (57.9)	5473.0 (77.8)	3.2% (1.0% to 5.3%)**	-0.3% (-0.5% to -0.03%)*
Emergency admissions	467.7 (5.7)	482.8 (4.9)	-5.5% (-6.6% to -4.3%)***	-0.4% (-0.9% to 0.03%)
In-hospital deaths	3.2 (0.1)	3.2 (0.1)	1.3% (-7.0% to 9.5%)	-0.4% (-0.6% to -0.2%)***

Abbreviation: SD, standard deviation; ED, emergency department; GOP, general outpatient.

a. Estimated changes were based on generalized linear regression models with log link, with district-months as the unit of analysis, assessing the pre-post level change in outcomes and the slope change after ED charge increase while accounting for the trend of preintervention period. The models accounted for district-level factors of sex, age groups (<15, 15-24, 25-44, 45-64 years), median income level, and calendar months and Fourier terms to account for seasonality in outcomes. Robust SEs were used to account for the clustering of repeated monthly measures at the district level.

***, *P*-value <0.001; **, *P*-value <0.01; *, *P*-value <0.05.

© 2023 Wu Y et al. *JAMA Network Open.*

eTable 4. Sensitivity Analysis Restricting Samples to 3 Lowest- and Highest-Income Districts[a]

	Monthly mean, per 100 000 population (SD)		Percentage changes, after vs before June 2017	
	June 2015-May 2017	June 2017 - May 2019	Level Change[a] (95% CI)	Slope Change[a] (95% CI)
**Low-income groups**				
Total ED visits	2123.5 (19.6)	1939.6 (21.4)	-6.0% (-7.7% to -4.4%)***	0.1% (-0.1% to 0.4%)
Emergency	50.2 (0.8)	54.8 (0.8)	-3.2% (-9.2% to 2.8%)	0.1% (-0.2% to 0.4%)
Urgent	590.9 (4.8)	577.2 (6.8)	-5.7% (-6.9% to -4.5%)***	0.2% (0.01% to 0.4%)
Non-urgent	1482.5 (15.2)	1307.8 (15.2)	-5.4% (-7.1% to -3.8%)***	0.2% (-0.1% to 0.4%)
GOP visits	7185.8 (79.6)	6952.3 (106.0)	4.8% (2.1% to 7.5%)**	-0.4% (-0.7% to -0.1%)**
Emergency admissions	529.7 (6.3)	549.8 (6.4)	-7.2% (-10.1% to -4.2%)***	0.2% (0.03% to 0.3%)*
In-hospital deaths	10.2 (0.3)	9.8 (0.2)	-8.5% (-20.9% to 3.9%)	0.3% (-0.1% to 0.7%)
**High-income groups**				
Total ED visits	1506.2 (13.0)	1366.2 (12.5)	-6.5% (-10.9% to -2.1%)**	0.3% (-0.3% to 0.8%)
Emergency	41.3 (0.7)	43.9 (0.8)	6.0% (3.9% to 8.2%)	0.6% (-0.3% to 0.9%)
Urgent	326.6 (3.1)	330.0 (3.4)	-5.9% (-9.0% to -2.9%)***	0.6% (0.3% to 0.9%)***
Non-urgent	1140.8 (12.0)	994.2 (10.4)	-6.5% (-8.2% to -4.8%)***	0.6% (0.3% to 0.9%)***
GOP visits	4188.5 (48.6)	3979.7 (63.0)	4.6% (2.6% to 6.6%)***	-0.2% (-0.7% to 0.3%)
Emergency admissions	383.4 (5.7)	386.7 (3.5)	-7.6% (-13.5% to -1.7%)*	0.2% (-0.2% to 0.6%)
In-hospital deaths	10.8 (0.3)	12.2 (0.4)	14.0% (-6.0% to 34.0%)	0.6% (0.5% to 0.8%)***

Abbreviation: SD, standard deviation; ED, emergency department; GOP, general outpatient.

a. Non-CSSA people were categorized into three income groups based on the median household income in their respective districts of residence. We rank districts by the median income level in 2017 and evenly classified into three groups
b. Estimated changes were based on generalized linear regression models with log link, with district-months as the unit of analysis, assessing the pre-post level change in outcomes and the slope change after ED charge increase while accounting for the trend of preintervention period. The models accounted for district-level factors of sex, age groups (<15, 15-24, 25-44, 45-64 years), median income level, and calendar months and Fourier terms to account for seasonality in outcomes. Robust SEs were used to account for the clustering of repeated monthly measures at the district level.

***, *P*-value <0.001; **, *P*-value <0.01; *, *P*-value <0.05.

© 2023 Wu Y et al. *JAMA Network Open.*

eTable 5 Placebo Analysis of Changes in Outcomes Among Comprehensive Social Security Assistance (CSSA) Patients

	Monthly mean, per 100 000 population (SD)		Percentage changes, after vs before June 2017	
	June 2015- May 2017	June 2017 - May 2019	Level Change[a] (95% CI)	Slope Change[a] (95% CI)
Total ED visits	7696.8 (106.7)	8002.5 (115.0)	1.9% (-0.4% to 4.3%)	-0.04% (-0.3% to 0.2%)
Emergency	333.1 (5.6)	388.6 (5.9)	2.1% (-6.1% to 10.4%)	-0.3% (-0.9% to 0.2%)
Urgent	2153.5 (26.9)	2370.1 (28.5)	-1.2% (-4.3% to 1.9%)	-0.2% (-0.6% to 0.3%)
Non-urgent	5309.6 (86.4)	5352.6 (88.3)	2.2% (2.4% to 6.9%)	-0.02% (-0.2% to 0.1%)
GOP visits	15944.6 (197.4)	15276.9 (227.7)	4.9% (3.2% to 6.6%)***	-0.6% (-0.9% to -0.4%)***
Emergency admissions	2043.9 (29.5)	2307.8 (26.9)	-0.2% (-3.9% to 3.7%)	-0.4 (-0.9% to 0.1%)
In-hospital deaths	161.5 (4.4)	177.3 (3.6)	-1.6% (-4.8% to 1.6%)	-0.2% (-0.5% to 0.3%)

Abbreviation: SD, standard deviation; ED, emergency department; GOP, general outpatient.

a. Estimated changes were based on generalized linear regression models with log link, with district-months as the unit of analysis, assessing the pre-post level change in outcomes and the slope change after ED charge increase while accounting for the trend of preintervention period. The models accounted for district-level factors of sex, age groups (<15, 15-24, 25-44, 45-64 years), median income level, and calendar months and Fourier terms to account for seasonality in outcomes. Robust SEs were used to account for the clustering of repeated monthly measures at the district level.

***, *P*-value <0.001; **, *P*-value <0.01; *, *P*-value <0.05.

© 2023 Wu Y et al. *JAMA Network Open.*

eTable 6 Characteristics of Comprehensive Social Security Assistance (CSSA) Recipients and Low-Income Non-CSSA People Visiting Emergency Departments in Public Hospitals, 2015-2019[a]

	CSSA recipients (n = 732 389)	Low-income non-CSSA people (n = 2 388 368)
**Sex**		
Male	362 740 (49.5%)	1 167 080 (48.8%)
Female	369 649 (50.5%)	1 221 289 (51.2%)
**Age**		
0–14	176 139 (24.0%)	420 328 (17.6%)
15–24	75 932 (10.4%)	281 963 (11.8%)
25–44	166 516 (22.7%)	744 997 (31.2%)
45–64	313 802 (42.8%)	941 081 (39.4%)
**District-level income levels**		
High-income	113 248 (15.5%)	
Middle-income	229 617 (31.4%)	
Low-income	389 524 (53.2%)	2 388 368 (100%)
**Triage category of ED visits**		
Emergency condition	25 010 (3.4%)	61 741 (2.6%)
Urgent condition	209 270 (28.6%)	640 488 (26.8%)
Non-urgent condition	498 110 (68.0%)	1 686 139 (70.6%)

a. Comprehensive social security assistance (CSSA) recipients are exempted from paying for public healthcare services. Non-CSSA people were categorized into three income groups based on the median household income in their respective districts of residence. We rank districts by the median income level in 2017 and evenly classified into three groups

© 2023 Wu Y et al. *JAMA Network Open.*

eTable 7 Difference-in-Difference Analysis of the Changes in Outcomes Among Low-Income Groups, Using Comprehensive Social Security Assistance (CSSA) Patients as Control Group

	**Percentage changes, after vs before June 2017**
	**Level Change[a] (95% CI)**
**Total ED visits**	-8.0% (-10.1% to -6.0%)***
Emergency	5.7% （-20.1% to 8.7%)
Urgent	-6.4% (-9.4% to -3.4%)***
Non-urgent	-8.5% (-13.5% to -3.5%)**
**GOP visits**	4.8% (-1.2% to 10.8%)
**Emergency admissions**	-4.8% (-7.5% to -2.2%)***
**In-hospital deaths**	-5.1% (-10.3% to 0.2%)

Abbreviation: SD, standard deviation; ED, emergency department; GOP, general outpatient.

a. Estimated changes were based on generalized linear regression models with log link, with district-months as the unit of analysis, assessing change in outcomes before and after ED charge increase using CSSA recipients as control group while accounting for the trend of preintervention period. The models accounted for district-level factors of sex, age groups (<15, 15-24, 25-44, 45-64 years), median income level, and calendar months and Fourier terms to account for seasonality in outcomes. Robust SEs were used to account for the clustering of repeated monthly measures at the district level.

***, *P*-value <0.001; **, *P*-value <0.01; *, *P*-value <0.05.

© 2023 Wu Y et al. *JAMA Network Open.*

eTable 8 Placebo Analysis of Changes in Nonemergency Admissions and Specialist Outpatient Visits Before and After ED Charge Increase

	Monthly mean, per 100 000 population (SD)		Percentage changes, after vs before June 2017	
	June 2015-May 2017	June 2017 - May 2019	Level Change[a] (95% CI)	Slope Change[a] (95% CI)
Non-Emergency admissions	645.8 (5.6)	660.2 (6.7)	2.2% (-5.4% to 1.1%)	-0.5% (-0.2% to 0.1%)
Specialist outpatient visits	5704 (64.4)	5770.6 (86.8)	-0.1% (-3.8% to 2.0%)	-0.05% (-0.25% to 0.14%)

Abbreviation: SD, standard deviation; ED, emergency department.

a. Estimated changes were based on generalized linear regression models with log link, with district-months as the unit of analysis, assessing the pre-post level change in outcomes and the slope change after ED charge increase while accounting for the trend of preintervention period. The models accounted for district-level factors of sex, age groups (<15, 15-24, 25-44, 45-64 years), median income level, and calendar months and Fourier terms to account for seasonality in outcomes. Robust SEs were used to account for the clustering of repeated monthly measures at the district level.

***, *P*-value <0.001; **, *P*-value <0.01; *, *P*-value <0.05.

© 2023 Wu Y et al. *JAMA Network Open.*
