# Supplementary material for: Emergency Department Use Across Income Groups Following an Increase in Cost-Sharing
Source: JAMA Netw Open. 2023 Aug 17;6(8):e2329577. doi: 10.1001/jamanetworkopen.2023.29577 (PMC10436128; doi:10.1001/jamanetworkopen.2023.29577)
Supplement: Supplement 2. — Data Sharing Statement [file jamanetwopen-e2329577-s002.pdf]

Data Sharing Statement

Wu. Emergency Department Use Across Income Groups Following an Increase in Cost-Sharing. *JAMA Netw Open*. Published August 17, 2023.
doi:10.1001/jamanetworkopen.2023.29577

Data
Data available: No

Additional Information
Explanation for why data not available: The government institution where we retrived the data does not allow us to make the data available to the public.
